# How is disease severity associated with quality of life in psoriasis patients? Evidence from a longitudinal population-based study in Sweden

**DOI:** 10.1186/s12955-017-0721-x

**Published:** 2017-07-28

**Authors:** Kirk Geale, Martin Henriksson, Marcus Schmitt-Egenolf

**Affiliations:** 1 Department of Public Health and Clinical Medicine, Dermatology, Umeå University, Umeå, Sweden; 2 PAREXEL International, Stockholm, Sweden; 3 Department of Medical and Health Sciences, Linköping University, Linköping, Sweden

**Keywords:** Quality of life, EQ-5D, Disease severity, Population-based data, Register data, Psoriasis

## Abstract

**Background:**

Assessing the impact of disease severity on generic quality of life (QOL) is a critical step in outcomes research and in the development of decision-analytic models structured around health states defined by clinical measures. While data from routine clinical practice found in healthcare registers are increasingly used for research, more attention should be paid to understanding the relationship between clinical measures of disease severity and QOL. The purpose of this work was therefore to investigate this relationship in psoriasis using a population-based dataset.

**Methods:**

Severity was measured by the Psoriasis Area and Severity Index (PASI), which combines severity of erythema, induration, and desquamation into a single value ranging from 0 to 72. The generic EQ-5D-3L utility instrument, under the UK tariff, was used to measure QOL. The association between PASI and EQ-5D-3L was estimated using a population-based dataset of 2674 patients with moderate to severe psoriasis enrolled over ten years in the Swedish psoriasis register (PsoReg). Given the repeated measurement of patients in the register data, a longitudinal fixed-effects model was employed to control for unobserved patient-level heterogeneity.

**Results:**

Marginal changes in PASI are associated with a non-linear response in EQ-5D-3L: Moving from PASI 10 to 9 (1 to 0) is associated with an increase of 0.0135 (0.0174) in EQ-5D-3L. Furthermore, unobserved patient-level heterogeneity appears to be an important source of confounding when estimating the relationship between QOL and PASI.

**Conclusions:**

Using register data to estimate the impact of disease severity on QOL while controlling for unobserved patient-level heterogeneity shows that PASI appears to have a larger impact on QOL than previously estimated. Routine collection of generic QOL data in registers should be encouraged to enable similar applications in other disease areas.

**Trial registration:**

Not applicable.

## Background

Economic evaluations of health technologies compare the efficiency of a new health intervention with one or more alternatives in cost-effectiveness analyses with quality-adjusted life years (QALYs) as outcomes. In order to be relevant for decision making clinical efficacy, generic quality of life (QOL), and cost data are often synthesized in a decision-analytic modelling framework [1]. Decision-analytic models used to evaluate cost-effectiveness are often based on clinically-defined health states, each of which has a cost and QOL profile [2]. Although establishing the relationship between measures of clinically-defined disease severity and QOL measures is an essential part of an economic evaluation, real-world datasets such as registers are underutilised and the methodologies employed often admit avoidable sources of bias.

Register data have increasingly been used to develop inputs for decision-analytic models, although deriving healthcare resource use and associated costs has likely been the most common research objective [3]. In Sweden, many population-based registers are available that include both disease severity and QOL measures, while reflecting actual clinical practice. A recent review shows that out of 103 Swedish healthcare registers, all include measures of disease severity, 46 measure QOL with EQ-5D, and 14 measure QOL with SF-36 [4]. Using unique personal identification numbers, these registers can be linked to other databases containing demographic, medical, and socioeconomic data.

Registers also provide follow up over time, making it possible to exploit the advantages of longitudinal statistical models. A primary advantage of a longitudinal approach is that time trends can be identified, allowing for observation of otherwise hidden relationships.

The objective of this study was to show how register data can be used to provide longitudinal estimates of the association between QOL and disease severity. This was illustrated using a case study in psoriasis, where similar estimates do not currently appear to be available.

## Methods

### Case study in psoriasis

Psoriasis is a chronic autoimmune disease affecting approximately 2–4% of the population in Western countries [5], and can be a debilitating disease that has negative consequences for patient QOL, due to physical and psychosocial factors. In psoriasis, disease severity is most often measured using the Psoriasis Area and Severity Index [6] (PASI), a multi-dimension questionnaire completed by the caregiver. This tool divides the body into four areas (head, arms, trunk, legs) where each area has an associated importance weight. The areas are scored individually for severity, measured by levels of erythema, induration, and desquamation, resulting in a score ranging from 0 (minimal severity) to a theoretical 72 (maximal severity). Economic evaluations commonly measure QOL using EQ-5D, a multi-attribute utility instrument with five dimensions [7]. The version with three levels of response was used in this study (EQ-5D-3L). Respondents fill in whether they have no, some, or extreme problems in each of the five dimensions (mobility, self-care, usual activities, pain/discomfort, and anxiety/depression). Patients’ responses were mapped to a single index value using the UK population value set [8], where the values are bounded between −0.59 and 1. A sensitivity analysis was conducted using the Swedish experience based value set, ranging from 0.34 to 0.97 [9].

Previous research on the association between PASI and EQ-5D-3L is scarce. Existing results are limited in terms of their ability to control for confounding variables [10–12], and have not controlled for unobserved heterogeneity such as genetic factors. Since there are unobserved variables that are correlated with both QOL and disease severity, estimates of the impact of disease severity could be biased by omitting these unmeasured factors.

### Overall analytic approach and data

Longitudinal fixed effects modelling is one technique to reduce bias arising from certain types of unobserved variables by using the patient as her own control. This method is recommended for certain types of data structures and research questions such as the ones in this study [13] as it exploits the longitudinal nature of the register data, as described in detail in the statistical methods section.

PsoReg is Sweden’s national psoriasis register, including patients with moderate to severe psoriasis who are eligible for systemic treatment from a specialist in dermatology [14]. Patients are registered on a continuous basis, where the current data cut includes patients registered from 2006 to 2014, including 4449 unique patients. The database contains disease-related data including severity and QOL, ideally recorded at each examination, and has been estimated to contain 65% of all psoriasis patients in Sweden receiving biologic treatment.

**Fig. 1:**
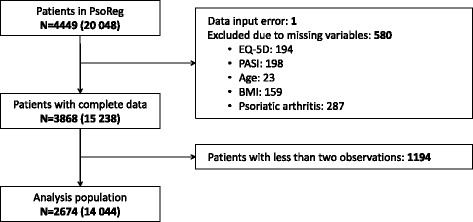
Dataset reduction diagram. A total of 4449 patients with 20,048 observations were present in PsoReg at the time of the data extraction. One patient was excluded due to an illogical height/weight value, and another 580 patients were removed due to missing covariate data resulting in 3868 patients with a total of 15,238 observations. In order to be included in the longitudinal analysis, at least two observations were required. 1194 did not meet this requirement. The final analysis population contained 2674 patients with a total of 14,044 observations

From the initial patient population of 4449 patients (20,048 observations), observations with missing data or input errors were excluded (Fig. 1). Patients with only one observation were also excluded, since the statistical methodology described below requires two or more observations per patient. In total, 2674 patients and 14,044 observations were included in the analysis with an average of 5.3 follow-up visits (minimum of 2, maximum of 36). The characteristics of patients and their disease in the analysis population (measured at patients’ first observation in PsoReg) and in the full PsoReg population are similar (Table 1).

**Table 1:** Patient and disease characteristics at first observation in PsoReg

Characteristic	Summary statistics	
	PsoReg population (*n* = 4449)	Analysis population (*n* = 2674)
EQ-5D		
n (missing)	4020 (429)	2674 (0)
Mean (SD)	0.71 (0.26)	0.73 (0.26)
Median (Q1; Q3)	0.73 (0.66; 0.85)	0.76 (0.69; 0.85)
Min; Max	−0.59; 1.00	−0.35; 1.00
PASI		
n (missing)	4061 (389)	2674 (0)
Mean (SD)	8.32 (7.01)	7.97 (6.74)
Median (Q1; Q3)	6.60 (3.20; 11.50)	6.30 (3.10; 10.00)
Min; Max	0.00; 67.80	0.00; 67.80
Age		
n (missing)	4423 (26)	2674 (0)
Mean (SD)	51.46 (15.14)	51.45 (14.77)
Median (Q1; Q3)	52.49 (40.79; 63.07)	52.44 (40.89; 62.60)
Min; Max	0.93; 91.73	9.43; 91.73
Gender		
n (missing)	4446 (3)	2674 (0)
Female	1804 (41%)	1069 (40%)
Male	2642 (59%)	1605 (60%)
BMI (kg/m^2^)		
n (missing)	4216 (233)	2674 (0)
Mean (SD)	27.89 (6.22)	27.86 (5.29)
Median (Q1; Q3)	27.10 (24.20; 30.70)	27.10 (24.20; 30.80)
Min; Max	14.20; 189.10	14.20; 59.50
Smoking status		
n (missing)	4423 (26)	2674 (0)
Smoker	1159 (26%)	725 (27%)
Non-smoker	3264 (74%)	1949 (73%)
Psoriatic arthritis		
n (missing)	3938 (511)	2674 (0)
No	2821 (72%)	1910 (71%)
Yes	1117 (28%)	764 (29%)

### Statistical methods

Quality of life is a complex phenomenon that is influenced by various factors, many of which are not observable. However, the fact that they are unobservable does not diminish their effect, and in order to reduce bias in the estimation of the impact of PASI on QOL those unobservable factors should be controlled for. As PsoReg data is longitudinal, analyses can address confounding due to time-invariant, patient-level, unobserved characteristics. Specifically, there are aspects of each patient, such as genetics, personality, and sex that do not tend to change over time. The first two are typically unobserved. While traditional models can control for observed time-invariant characteristics such as sex, more sophisticated (longitudinal) models, such as fixed or random effects models, can also account for unobserved time-invariant effects.

The estimation of the treatment effect is more efficient in a random-effects model than a fixed-effect model, but the former makes the assumption that the unobserved individual level effects are uncorrelated with the observed variables. Fixed and random effects models are both unbiased when this assumption holds, but only fixed effects are unbiased when it does not. In the context of this analysis, independence between the observed and unobserved effects is unreasonable. For example, since genetics (unobserved fixed effect) predispose certain people to psoriatic arthritis (observed effect), and both are correlated with QOL, the independence assumption does not hold and a fixed-effects model should be estimated. Although fixed effects methods used in this analysis have been well described in statistical literature, different definitions of the term “fixed effects” are sometimes used. For clarity, the theoretical model is written in Eqs. (1), (2), (3).

1 $$ {y}_{i,t}={X}_{i,t}\beta +{\delta}_i+{e}_{i,t.} $$

2 $$ {y}_{i,t}-{\overline{y}}_i=\left({X}_{i,t}-{\overline{X}}_i\right)\beta +\left({\delta}_i-{\overline{\delta}}_i\right)+\left({e}_{i,t}-{\overline{e}}_i\right),\kern0.5em  where\ {\overline{z}}_i=\frac{1}{T}{\sum}_t^T{Z}_{i,t}\ for\ \mathrm{any}\;\mathrm{variable}\ z $$

3 $$ {\ddot{y}}_{i,t}={\ddot{X}}_{i,t}\beta +{\ddot{e}}_{i,t} $$

The index i is the patient and t is the time index. y is QOL, X is the matrix of observed variables, δ is the vector of unobserved individual fixed effects, and e is the random error component. The model in Eq. (3) is actually estimated, and provides equivalent estimates of β as in Eq. (1) according to the Frisch-Waugh-Lovell theorem [15, 16]. Eq. (1) cannot be estimated because the fixed effects (δ_i) are not observed. The double dot accent (Eq. 3) represents the value of each variable after subtracting the average over all observations for each patient (Eq. 2).

In the analysis, the dependent variable is QOL, measured by EQ-5D-3L. The quadratic term of each variable was tested for influence in the estimated model by minimising the Akaike information criteria (AIC). The model with the lowest AIC (−12,930) included quadratic PASI and BMI terms and therefore these were included in the model. Interaction terms between PASI and age, PASI and psoriatic arthritis, and age and psoriatic arthritis were also included. The fixed effects estimator used a constraint that the sum of the individual fixed effects equal 0, implying that the intercept is calculated so that the predicted values of EQ-5D-3L equal the average value of EQ-5D-3L at the mean of each variable.

To confirm the necessity of using fixed effects instead of random effects as described above, a Hausman test [17] was performed which rejected the null hypothesis that both estimators are consistent, at an alpha level of 0.01. As such, the final model used fixed effects. Robust standard errors were also used following a Wald test for group-wise homoscedasticity [18], which was rejected at an alpha level of 0.01.

Stata v11.2 (*www.stata.com*) was used to analyse data and R version 3.1.1 (*www.r-project.org*) for graphics.

## Results

The increase in EQ-5D-3L due to the decrease in PASI depends on the value of PASI and its square as well as the interactions with age and PSA. The linear and quadratic effects of PASI are both statistically significant (*p* < 0.01). The interaction of PASI with age is also significant (*p* < 0.01) while the interaction with PSA is significant at a 10% level (*p* = 0.08) (Table 2).

**Table 2:** Regression results

Variable	Excluding fixed effects		Including fixed effects	
	Coefficient	95% CI	Coefficient	95% CI
Constant	0.9437 ***	0.8893, 0.9982	1.1764 ***	1.0027, 1.3500
PASI	−0.0186 ***	0.0000, 0.0002	−0.0224 ***	−0.0267, −0.0181
PASI squared	0.0001 ***	−0.0017, −0.0003	0.0002 ***	0.0001, 0.0003
Age	−0.0010 ***	−0.0035, −0.0003	−0.0057 ***	−0.0087, −0.0026
BMI	−0.0019 **	0.0000, 0.0000	−0.0003	−0.0031, 0.0026
BMI squared	−2.2 × 10^−05^ ***	−3.3 × 10^−05^, −9.91 × 10^−06^	1.99 × 10^−05^ ***	−2.57 × 10^−05^, −1.40 × 10^−05^
Psoriatic arthritis	−0.0570 *	0.0000, 0.0002	−0.0767	−0.1920, 0.0385
PASI * Age	0.0001 **	−0.0055, 0.0001	0.0001***	0.0000, 0.0002
PASI * Psoriatic arthritis	−0.0027 *	−0.0013, 0.0010	−0.0021*	−0.0045, 0.0002
Age * Psoriatic arthritis	−0.0001	0.0527, 0.0849	0.0015	−0.0006, 0.0035
Male	0.0688 ***	0.8893, 0.9982		

*p < 0.10, **p < 0.05, ***p < 0.01; CI: Confidence interval; Patients: 2674; Observations: 14,044

R^2^ = 0.612, Adjusted R^2^ = 0.520

**Fig. 2:**
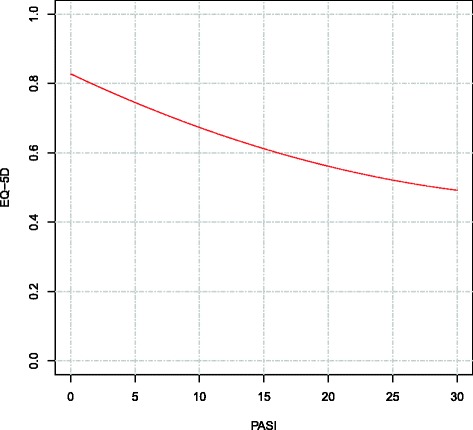
Predicted EQ-5D-3L values given marginal changes in PASI. Using the results of the fixed-effects regression model, PASI was varied from 0 to 30, showing the predicted marginal impact on EQ-5D-3L. Although PASI can theoretically range from 0 to 72, a cut-off of 30 was chosen based on the 99th percentile of observed PASI scores, equal to 25.1. All other variables in the model were held constant at their respective averages. The curve shows the non-linear relationship between PASI and EQ-5D-3L, where increases in PASI close to 0 have a larger impact on EQ-5D-3L than an equally-sized increase in PASI at higher ends of the PASI scale

**Fig. 3:**
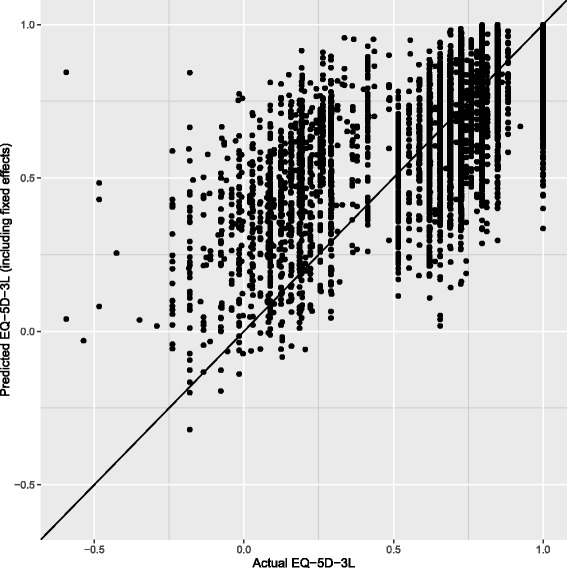
Actual vs. predicted values of EQ-5D-3L. This model diagnostic figure shows the relationship between the actual observed EQ-5D-3L values compared to the values predicted by the fixed-effects regression model. The 45-degree line indicates where the predicted and actual EQ-5D-3L values are equal. The figure shows that for low actual EQ-5D-3L values, the regression model may overestimate EQ-5D-3L, and for high actual EQ-5D-3L values, the model may under-estimate EQ-5D-3L. Overall, the model predicts EQ-5D-3L with a relatively high level of accuracy

Based on the regression results, EQ-5D-3L was predicted as a function of PASI holding all other variables constant at their respective mean (Fig. 2 and Table 3). The x-axis in Fig. 3 extends to where PASI is equal to 30; the 99th percentile of the PASI values in PsoReg is 25.1.

**Table 3:** Predicted EQ-5D-3L for varying levels of PASI

PASI	Predicted EQ-5D-3L	Difference from PASI = 0 (disutility)
0	0.8280	N/A
1	0.8106	0.0174
2	0.7937	0.0343
3	0.7772	0.0509
4	0.7610	0.0670
5	0.7454	0.0827
10	0.6734	0.1547
15	0.6121	0.2160
20	0.5614	0.2666
25	0.5215	0.3066
30	0.4922	0.3359

The model explained 61.2% of variance in EQ-5D-3L according to the R^2^ measure of goodness of fit. Within the sample, the individual fixed effects can be calculated. In this case, the model’s (Eq. 1) root mean square error (RMSE) of the actual EQ-5D-3L value compared to the model’s prediction was 0.152 and the mean absolute error (MAE) was 0.104 (Fig. 3).

As discussed in the methods, the fixed effects regression cannot estimate the effects of time-invariant effect such as sex. However, the effect of PASI on EQ-5D-3L can be calculated within sex stratifications (Table 4), where it appears that the direct linear effect of PASI is stronger in females than males.

**Table 4:** Fixed-effect regression results (sex stratification)

	Females		Males	
Variable	Coefficient	95% CI	Coefficient	95% CI
Constant	1.2778 ***	1.0068, 1.5488	0.7011 ***	0.2850, 1.1171
PASI	−0.0281 ***	−0.0360, −0.0201	−0.0206 ***	−0.0255, −0.0157
PASI squared	0.0003 ***	0.0001, 0.0004	0.0002 ***	0.0001, 0.0003
Age	−0.0078 ***	−0.0126, −0.0030	−0.0046 **	−0.0085, −0.0007
BMI	−0.0002 **	−0.0039, 0.0036	0.0282 **	0.0062, 0.0501
BMI squared	−1.8 × 10^−05^ ***	−2 × 10^−05^, −1.5 × 10^−05^	−0.0005 ***	−0.0008, −0.0001
Psoriatic arthritis	−0.2795 **	−0.4940, −0.0650	0.0206	−0.0898, 0.1310
PASI * Age	0.0001	0.0000, 0.0002	0.0001 ***	0.0000, 0.0002
PASI * Psoriatic arthritis	0.0034	−0.0008, 0.0077	−0.0046 ***	−0.0073, −0.0019
Age * Psoriatic arthritis	0.0046 **	0.0010, 0.0081	−0.0002	−0.0022, 0.0019
Patients (observations)	1069 (5392)		1605 (8652)	

*p < 0.10, **p < 0.05, ***p < 0.01; *CI* Confidence interval

The results described above use the UK EQ-5D-3L tariff. A sensitivity analysis was conducted using the Swedish EQ-5D-3L tariff, which has a smaller possible range of values. Using the same analysis as above where the only change was to replace the UK-based EQ-5D-3L index value with the Swedish EQ-5D-3L index value, the coefficient of the linear PASI estimate was −0.0086 (95% CI -0.0104, −0.0069; *p* < 0.001). The estimate of the PASI squared estimate was positive and statistically significant, but very small. The resulting R^2^ using the Swedish tariff was 0.665.

## Discussion

Developing QOL inputs for economic evaluations, based on clinical definitions of disease severity, is a core component of an economic assessment. In practice, this is often hindered by a lack of available data and methodology that allows researchers to address selection bias, obtain relevant covariates, and observe patients for a sufficiently long duration. This study illustrates how register data can be used to derive the association between disease severity and QOL by exploiting the unique content and structure of registers. Registers contain many relevant clinical and QOL variables, and may be linked to other databases containing sociodemographic information, allowing for adjustment of many sources of confounding. Even when an important confounding variable is not available, the longitudinal data structure can be exploited to control for factors that do not change within patients over time, such as genetics. Adjusting for all relevant factors, observed or unobserved, is one of the most important steps in reducing bias in a statistical analysis, which can be accomplished by employing the methods and type of data proposed in this study to estimate the association between QOL and disease severity. In turn, this may also reduce bias and uncertainty in economic evaluations where these estimates are used as parameter inputs.

In the case study presented here, the impact of longitudinal modelling methods in reducing bias can be observed by comparing predicted values where fixed effects are included or excluded: At PASI 10, all else equal, the predicted EQ-5D-3L controlling for fixed effects is 0.673, while excluding fixed effects is 0.708 (a difference of 0.035). In an economic evaluation, this relatively small difference in state-specific QOL may result in large differences when accumulated over time, particularly important when evaluating chronic diseases such as psoriasis, and thus have a large impact on the results of a cost-effectiveness analysis.

In terms of performance, the model’s predictive ability compared well to previous research. A meta-analysis of 119 models mapping disease specific measures to generic preference-based measures shows that previous studies have an R^2^ between 0.17–0.51, an MAE between 0.00–0.19, and an RMSE between 0.08–0.20 [19]. The predicted EQ-5D-3L values including fixed effects result in MAE and MRSE values that fall in the middle of this range. The R^2^ and adjusted R^2^ values of this study are above or at the top of this range, implying that the variation in the predictions from the estimated model is comparable to other mapping-focused research, and that the model explains more variation than most previous mappings.

In the sensitivity analysis using the Swedish EQ-5D-3L tariff, the finding that the PASI coefficient estimates using the Swedish tariff are smaller than under the UK tariff are unsurprising given the smaller range of the EQ-5D-3L index value using the Swedish tariff compared to the UK tariff. Interestingly, the linear PASI coefficient is approximately 2.6 times larger in the analysis using the UK tariff compared to the Swedish tariff, and the range of EQ-5D-3L values is also approximately 2.6 times larger in the UK tariff compared to the Swedish tariff. This may suggest that in some sense, the magnitude of the association between PASI and EQ-5D-3L is consistent in both analyses, where the difference may reflect the range of the dependent variable. The goodness of fit estimates suggest that the model may explain somewhat more variation in EQ-5D-3L under the Swedish tariff than the UK tariff.

In addition to illustrating how register data can generally be used to associate disease severity and QOL, this psoriasis case study also provides the first results of this association that control for unobserved patient characteristics in psoriasis. The results show that there is a statistically significant, negative, non-linear relationship between disease severity and QOL in psoriasis. Previous estimates of this relationship using PsoReg data provided estimates of the impact of PASI on QOL using EQ-5D-3L [11]. These results were not fully adjusted and used a less complex functional form, but it is the only study that estimated the association in a comparable way to our study, and the estimates show that each unit reduction in PASI results in a reduction of EQ-5D-3L by 0.0089 [11]. In the present study, changes in PASI result in non-linear changes in EQ-5D-3L. However, the size of the effect is larger than previously estimated for PASI values below 21: Moving from PASI 10 to 9 results in a marginal EQ-5D-3L change of 0.0135 and a move from PASI 1 to 0 results in an increase in EQ-5D-3L of 0.0174. At the upper end of the PASI scale, smaller changes in EQ-5D-3L measured QOL are predicted: moving from PASI 25 to 24 results in an increase in QOL of 0.0071, highlighting the non-linearity of the relationship. For low PASI scores, the impact of a marginal change in PASI on QOL is therefore greater than previously estimated.

The results can also be put into the context of typical clinical endpoints in psoriasis, which are often based on percentage PASI improvements (i.e. PASI 90). The change in absolute QOL can be calculated based on a patient’s baseline value and percentage improvement. For example, if it is known that a patient achieved 90% PASI improvement and started with PASI equal to 10, we can infer that patient improved to at least PASI equal to 1, a total of 9 units. Using the results of the regression, the value of EQ-5D-3L at PASI = 10 and PASI = 1 can be calculated (Table 3), where the difference is equal to 0.1372. This value represents the estimated difference in EQ-5D-3L between a PASI = 9 and PASI = 1 health state after controlling for each observed variable as well as time-invariant unobserved fixed effects.

An important aspect of decision-analytic modelling it to account for patient heterogeneity, as cost-effectiveness may differ in patients with different characteristics. This implies that input parameters, including QOL, should ideally be estimated for different subgroups of patients. The data and analytic approach employed in this study can be used to achieve this by disaggregating QOL based on applicable patient characteristics. For example, using the information derived in the study, it is possible to calculate the expected EQ-5D-3L specifically for a 60 year-old patient with PSA, a BMI of 30, and a PASI score of 10 (including the interactions). For a patient with these characteristics, the predicted EQ-5D-3L is 0.656 compared to the average aggregated patient, where predicted EQ-5D-3L is 0.673. As illustrated above, a benefit of the model estimated in this study is that it is a classic linear model, as it is linear in parameters while allowing for non-linear relationships between EQ-5D-3L and each regressor. This means that the coefficients estimated for each variable have the simple interpretation that a one unit increase in the variable value corresponds to a change in EQ-5D-3L equal to the size and direction of the coefficient.

Since fixed-effects models cannot estimate time-invariant parameters such as sex, stratification can be used to estimate the results in different patient groups. In the psoriasis case study, the results show that the effect of reducing PASI by one unit may have a larger impact on QOL for female patients than males (Table 4). Put in a clinical context, overall health gains in psoriasis patients may depend on baseline PASI score [20], treatment effect and the corresponding absolute change from baseline PASI [21, 22], and patient characteristics such as sex.

### Limitations and future research

Registers are a rich source of information, and we have illustrated how they can be utilized if they include both disease severity and QOL. However, they can vary in both content and quality, and it is therefore important to evaluate the usefulness and representativeness of the data on a case-by-case basis with respect to the percentage of eligible patients, potential for bias, and which measures of severity and QOL are available.

Provided that reliable data are available in registers, we believe that future research should employ the methods outlined in this work in order to estimate the association between QOL and clinical measures of disease severity in disease areas other than psoriasis. Differences in predicted QOL between various data sources, such as registers and trial data, could also be explored further from both a qualitative and quantitative perspective.

## Conclusions

Register data collected from routine clinical practice can be a valuable source of information to estimate the relationship between clinical measures of disease severity and QOL, a central component in economic evaluations. Given the data structure of registers, longitudinal modelling techniques provide a means to adjust for unobserved patient-level heterogeneity. In a case study combining register data with longitudinal modelling techniques, a non-linear relationship between clinically defined disease severity and QOL was found.

## Abbreviations

AIC: Akaike information criteria
EQ-5D-3L: 3-level EuroQoL 5-dimension instrument
PASI: Psoriasis Area and Severity Index
QALY: Quality-adjusted life years
QOL: (Generic) quality of life
